# First report of *Entamoeba moshkovskii* in human stool samples from symptomatic and asymptomatic participants in Kenya

**DOI:** 10.1186/s40794-019-0098-4

**Published:** 2019-12-17

**Authors:** Cecilia Kyany’a, Fredrick Eyase, Elizabeth Odundo, Erick Kipkirui, Nancy Kipkemoi, Ronald Kirera, Cliff Philip, Janet Ndonye, Mary Kirui, Abigael Ombogo, Margaret Koech, Wallace Bulimo, Christine E. Hulseberg

**Affiliations:** 1 United States Army Medical Research Directorate-Africa, P.O. Box 606-00621, Village Market, Nairobi, Kenya; 2 Jomo Kenyatta University of Science and Technology, P.O Box 62000-00200, Nairobi, Kenya; 3 United States Army Medical Research Institute of Infectious Diseases, Fort Detrick, Maryland USA

**Keywords:** Entamoeba, *Entamoeba moshkovskii*, Diarrhea, Kenya, Nested PCR

## Abstract

*Entamoeba moshkovskii* is a member of the *Entamoeba* complex and a colonizer of the human gut. We used nested polymerase chain reaction (PCR) to differentiate *Entamoeba* species in stool samples that had previously been screened by microscopy. Forty-six samples were tested, 23 of which had previously been identified as *Entamoeba* complex positive by microscopy. Of the 46 specimens tested, we identified nine (19.5%) as *E. moshkovskii-*positive. In seven of these nine *E. moshkovskii*-positive samples, either *E. dispar* or *E. histolytica* (or both) were also identified, suggesting that co-infections may be common. *E. moshkovskii* was also detected in both symptomatic and asymptomatic participants. To the best of our knowledge, this is the first report of *E. moshkovskii* in Kenya.

## Introduction

*Entamoeba moshkovskii* is a member of the *Entamoeba* complex and is morphologically indistinguishable from *E. dispar* and the pathogenic *E. histolytica*. WHO recommends treatment of both symptomatic (diarrheal) and asymptomatic (non-diarrheal) forms of *E. histolytica* infection only [1]. Metronidazole or tinidazole followed by iodoquinol or paromomycin is used for treatment of symptomatic *E. histolytica* infections whereas asymptomatic infections are treated using and iodoquinol or paromomycin [2]. Initially considered a free-living amoeba [3], there have been colonization reports of *E. moshkovskii* in humans over the years from Yemen [4], India [5], Indonesia, Colombia [6], Malaysia [7], Tunisia [8], Tanzania [9], and Australia [10]. Beck et al., [9] identified *E. moshkovskii* carriage in a Tanzanian population. In India, *E. moshkovskii* was reported not to cause diarrhea but as a cause of mild abdominal discomfort [5], while in Malaysia *E. moshkovskii* was isolated from both symptomatic and asymptomatic participants [7]. A 2012 study by Shimokawa and collegues [11] pointed to the possible pathogenicity of *E. moshkovskii* as a cause of diarrhea in mice and infants. Few studies have investigated the distribution of *E. moshkovskii* in Africa. We sought to screen for *E. moshkovskii* in stool samples from an ongoing surveillance study on enteric pathogens in Kenya using a PCR based assay.

## Materials and methods

The current study is a retrospective lab-based study nested in an ongoing case-control enteric surveillance study under the US Army Medical Research Directorate Africa at the Microbiology Hub. Archived stool samples collected between April 2013 and September 2014 from out-patient participants enrolled in a ‘Surveillance of Enteric Pathogens Causing Diarrheal Illness in Kenya’ study across seven participating public hospitals were analysed. Out-patients across all age-groups qualified to be enrolled as case participants (symptomatic) if they presented with 3–4 diarrheal episodes within 24 h and lasting less than 14 days. Out-patients presenting in the same hospitals with no diarrheal episodes within the last 14 days were enrolled as age-matched controls (asymptomatic). Stool aliquots for analysis of parasitic pathogens were suspended in the Mini Parasep® SF fecal parasite concentrator (Apacor, Wokingham, United Kingdom) and shipped at 2–8 °C. On arrival at the lab, the samples were centrifuged and a wet-preparation of the filtrate examined under light microscopy. A sample was reported as positive for the *Entamoeba* complex by either visualizing the trophozoites and/or spherical cysts with 1–4 nuclei. By microscopy, 23 samples were detected as positive for *Entamoeba* complex. These samples were then matched with their corresponding microscopy negative symptomatic or asymptomatic sample. A total of 46 specimens, 23 (6 symptomatic and 17 asymptomatic) previously identified as positive for *Entamoeba* complex by microscopy and their corresponding age-matched symptomatic or asymptomatic participants were screened by PCR for *Entamoeba* complex species. Of the total 46 samples tested 22 were from symptomatic and 24 from asymptomatic participants.

Samples were retrieved from long-term storage at − 80 °C and DNA extracted using the QIAmp DNA stool mini-kit®, (QIAGEN, Hilden, Germany) as per manufacturer’s instructions with slight modifications: incubation time with lysis buffer was 10 min at 95 °C, incubation time with InhibitEX was 10 min at room temperature, and incubation time with proteinase-K was 15 min at 70 °C. Species detection was carried out using a nested multiplex PCR previously described [12] using primers as listed below. Cycling conditions were as follows for the genus-specific PCR; 96 °C for 2 min followed by 30 cycles each consisting of 92 °C for 1 min, 56 °C for 1 min and 72 °C for 1 min. For the species-specific PCR, the cycling conditions were maintained and only the annealing temperature adjusted to 48 °C.

The DNA extracts from control strains of *E. dispar* (SAW 760), *E. moshkovskii* (Laredo) and *E. histolytica* (HM-1: IMSS) were a generous gift from Dr. Graham Clark of the London School of Tropical Medicine and Hygiene. Nuclease-free water was included as a negative control for each test run. The unknown samples and controls were run on an agarose gel, and amplicons of the seven samples with fragment sizes corresponding to *E. histolytica* (439 bp), five samples with fragments corresponding to *E. moshkovskii* (553 bp) and two samples with fragments corresponding to *E. dispar* (174 bp) were purified using ExoSAP-IT kit (ThermoFisher Scientific, Massachusetts, USA) kit and sequenced using standard capillary electrophoresis on a 3500xL Genetic Analyzer (Applied Biosystems, California, USA). The secondary PCR species-specific primers (Table 1) were used for sequencing. Chromatograms were visualized on Chromas and sequences analyzed using DNA sequence assembler v3, www.DnaBaser.com. Consensus sequences were compared to those on GenBank using BLASTn and sequences deposited into GenBank under accession numbers MK142734-MK142737*(E. moshkovskii)*, MH752550-MH752556 (*E. histolytica*) and MH754938, MH754939 (*E. dispar)*. Maximum likelihood phylogenetic relationships between the species were reconstructed using phyML 3.1 [13] employing the GTR + G model and 100 bootstraps. The tree was rooted to *Entamoeba coli* FR684433. Chi-square tests of association were performed to investigate possible associations between the presence of *E. moshkovskii* and symptomatic (cases) and/or asymptomatic (controls) infections.

**Table 1:** List of ribosomal 18S primers used in this study

Genus-specific primers (First PCR)	
*Entamoeba genus*	E-1 5' TAAGATGCACGAGAGCGAAA 3' (forward primer)
	E-2 5' GTACAAAGGGCAGGGACGTA 3' (reverse primer)
Species-specific primers (Second nested multiplex PCR)	
*E. histolytica species*	EH-1 5' AAGCATTGTTTCTAGATCTGAG 3' (forward primer)
	EH-2 5' AAGAGGTCTAACCGAAATTAG 3' (reverse primer)
*E. moshkovskii species*	EM-1 5' GAAACCAAGAGTTTCACAAC 3' (forward primer)
	EM-2 5' CAATATAAGGCTTGGATGAT 3' (reverse primer)
*E. dispar species*	ED-1 5' TCTAATTTCGATTAGAACTCT 3' (forward primer)
	ED-2 5' TCCCTACCTATTAGACATAGC 3' (reverse primer)

Ethical clearance for this work was obtained from Kenya Medical Research Institute Scientific and Ethical Review Unit (SERU-SSC) and Walter Reed Army Institute of Research (WRAIR) institutional review boards (IRBs) (SSC # 3365, WRAIR #1549B).

## Results

**Fig. 1:**
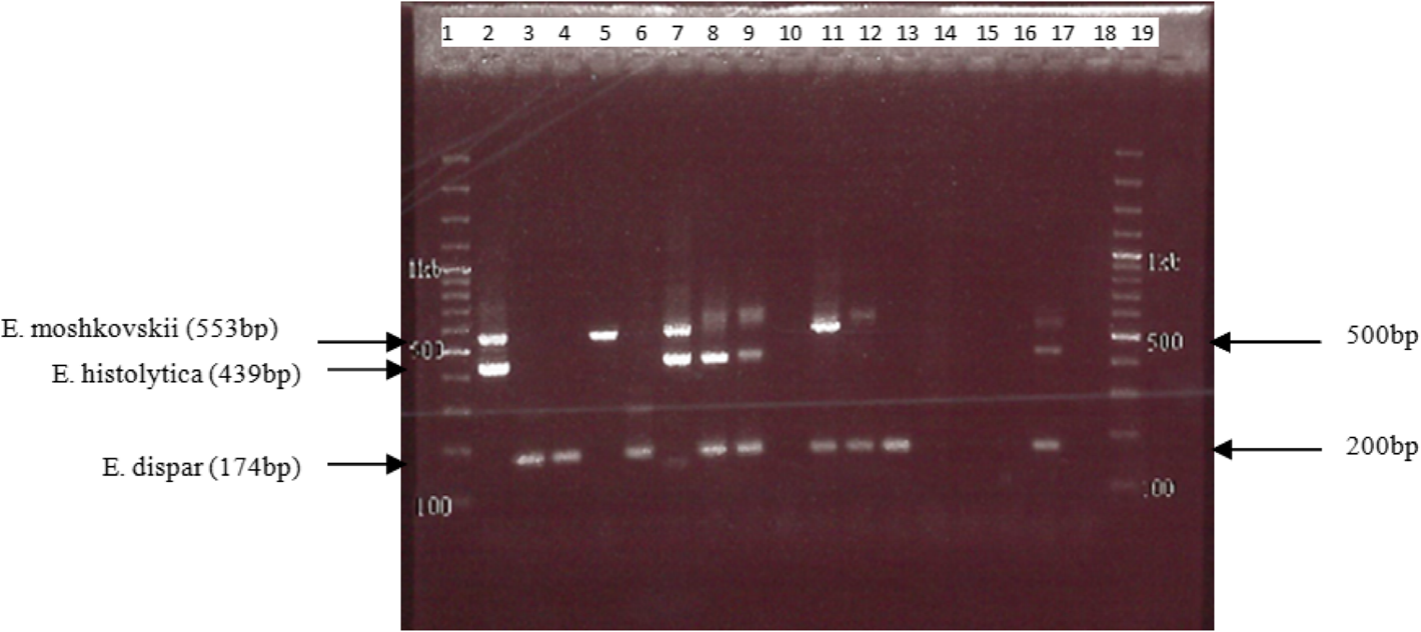
Species differentiation of *Entamoeba* complex species. Lane 1 and 19 are 100 bp molecular weight ladder. Lane 2- *E. histolytica* and *E. moshkovskii* co-infection, lane 3, 4 – *E. dispar* (mono-infection), lane 5- *E. moshkovskii* (mono-infection),lane 6 – *E. dispar* (mono-infection), lane 7- *E. histolytica* and *E. moshkovskii*, lane 8,9 - *E. dispar* and *E. histolytica*, lane 10 – negative, lane 11,12 – *E. dispar* and *E. moshkovskii*, lane 13 – *E. dispar*, lane 14, 15, 16 – negative, lane 17 – positive control (*E. dispar, E. histolytica, E. moshkovskii*), lane 18 – negative control

Out of the 46 samples tested by PCR, 22 (47.8%) were positive for *Entamoeba* complex. Of these, 16 had initially been identified as positive for *Entamoeba* complex by microscopy (Table 2). Among the 22 PCR-positives, *Entamoeba* complex species were identified as follows: nine were *E. dispar* (40.9%), two were *E. moshkovskii* (9.1%), and one was *E. histolytica* (4.5%)*.* Combinations of *Entamoeba* species detected were: three *E. histolytica* and *E .dispar* (13.6%), two *E. histolytica* + *E. moshkovskii* (9.1%), four *E. moshkovskii* and *E. dispar* (18.2%) and one *E. histolytica* and *E. dispar* and *E. moshkovskii* (Table 3) (Fig. 1).

**Table 2:** Agreement of *Entamoeba* species identification by PCR and microscopy

	Microscopy		Total samples
	Positive (*n* = 23)	Negative (n = 23)	
PCR			
Positive	16	6	22
Negative	7	17	24
Total	23	23	46

**Table 3:** Distribution of *Entamoeba* complex species identified by PCR

Microscopy	PCR		Disease status	
	Species	Number PCR positive (%)	Symptomatic (%)	Asymptomatic (%)
Positive (n = 23)	*E. dispar* only	9 (40.9)	3 (33)	6 (67)
	*E. moshkovskii* only	2 (9.1)	1 (50)	1 (50)
	*E. histolytica* + *E. dispar*	2 (9.1)	1 (50)	1 (50)
	*E. histolytica* + *E. moshkovskii*	2 (9.1)	0 (0)	2 (100)
	*E. dispar* + *E. moshkovskii*	1 (4.5)	0 (0)	1 (100)
Negative (n = 23)	*E. histolytica* only	1 (4.5)	1 (100)	0 (0)
	*E. histolytica* + *E. dispar*	1 (4.5)	0 (0)	1 (100)
	*E. dispar* + *E. moshkovskii*	3 (13.6)	2 (67)	1 (33)
	*E. histolytica* + *E. moshkovskii* + *E. dispar*	1 (4.5)	1 (100)	0 (0)

**Fig. 2:**
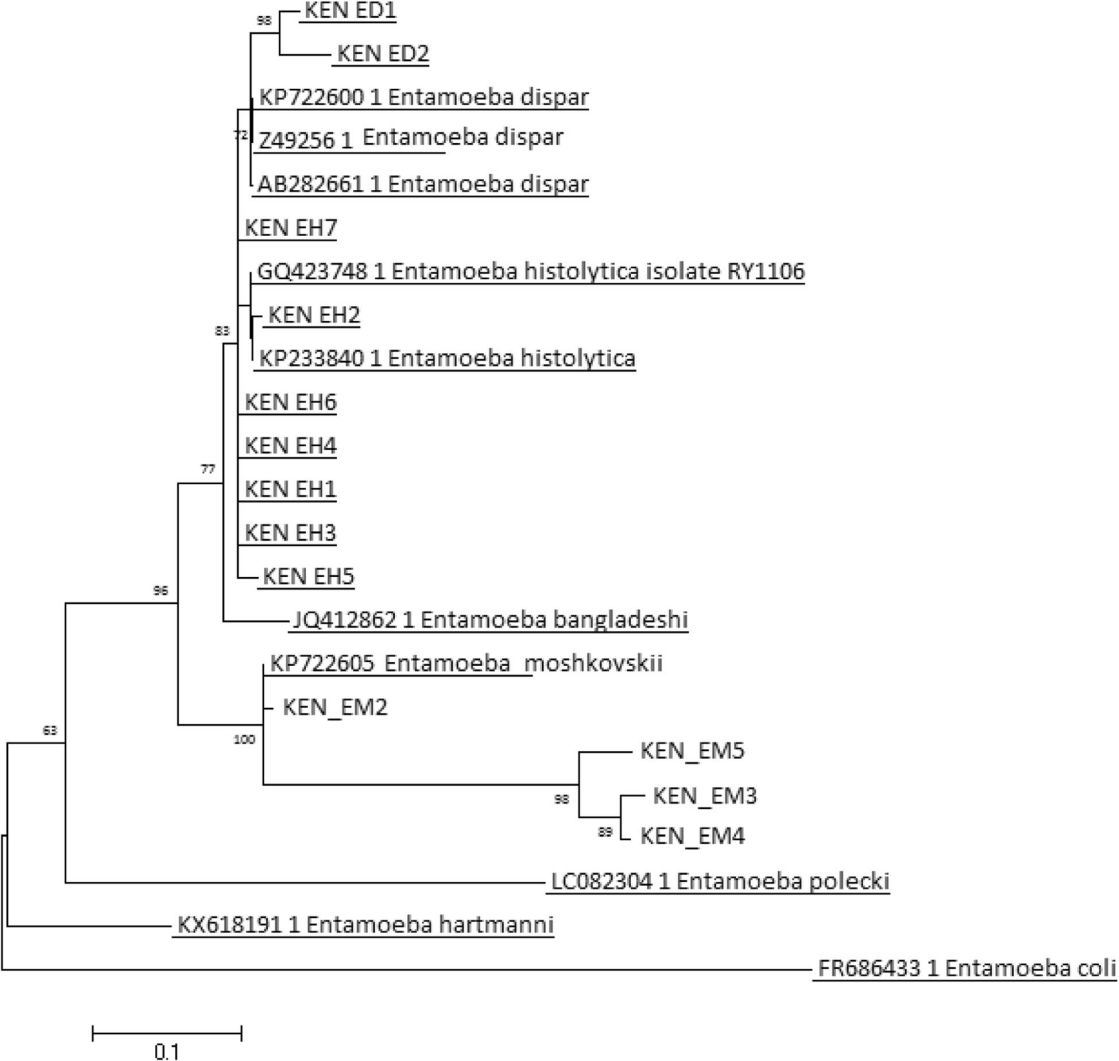
Phylogenetic reconstruction of *Entamoeba* complex species based upon 18S rRNA. Samples sequenced in this study were designated with a KEN prefix (EH: *E. histolytica*, ED: *E. dispar*, EM: *E. moshkovskii*) alongside reference sequences from GenBank. Four *E. moshkovskii* (KEN EM2 - KEN EM5) were identified in this study. Branch values show bootstrap support

*E. moshkovskii* mono-infection was identified in two samples and in seven samples as co-infection with either *E. dispar*, *E. histolytica* or both. Of the nine PCR-positive *E. moshkovskii* samples, five had previously been identified by microscopy as *Entamoeba* complex, while the remaining were missed identifications. *E. moshkovskii* was identified both in symptomatic and asymptomatic participants with no statistically significant differences. Of the nine bands corresponding to *E. moshkovskii* band size (553 bp), four were very faint and could not be sequenced. This could be attributed to possible low parasite copy number in the stool specimens. One sample failed to sequence thus sequence analysis was performed on only four high-quality bands. Sequence analysis of *E. moshkovskii* bands revealed 99% identity to the Laredo reference strain of *E. moshkovskii* (KP722605.1). Reconstruction of phylogenetic relationships revealed distinct species-specific clustering (Fig. 2).

## Discussion

This study speciated members of the *Entamoeba* complex from stool specimens using PCR. We then sequenced the *E. moshkovskii*-positive samples to confirm proper species identification and constructed a phylogenetic tree. To the best of our knowledge, this is the first survey of the distribution of *Entamoeba* species in clinical samples originating from Kenyan symptomatic and asymptomatic participants.

Although the number of samples tested in this study is fairly small, co-infections among species of the *Entamoeba* complex appear to be common. We also detected *E. moshkovskii* alongside *E. dispar* and *E. histolytica* and noted a high frequency of detection for *E. dispar*. This detection of *E. moshkovskii* alongside these two species is also consistent with other reports that *Entamoeba* species co-circulate in *Entamoeba* endemic areas [14]. Previous Kenyan studies [15–21] have largely focused on microscopic identification of *Entamoeba* complex and/or molecular identification of *E. histolytica.* This has left a gap in our understanding of the epidemiological distribution of *Entamoeba* species in Kenya. Paucity of differentiation studies has in addition denied important assessments as to whether they contribute to gastrointestinal disease in humans. A recent molecular epidemiological study of *Entamoeba* species, involving asymptomatic children from western Kenya, using a nested PCR assay, did not detect any *E. moshkovskii* infections [17]. Although Matey et al., did not detect *E. moshkovskii* in their study population, it is possible that *E. moshkovskii* infections have been in circulation in Kenya and are only now being identified.

This study focused on identifying three members of the *Entamoeba* complex thus it is possible that some of the microscopy misidentifications could be other members of the broader *Entamoeba* genus. For instance, cysts of *E. hartmanii* can be misidentified as *Entamoeba* complex since despite being relatively smaller, they too possess 1–4 nuclei. Furthermore, the missed identifications by microscopy are often attributable to a low number of cysts in the stool, degraded trophozoites, varying technical skill among technicians, human error and subjectivity.

Phylogenetic analyses showed *Entamoeba* complex spp. were closely related to but genetically distinct from other *Entamoeba* spp. (*E. coli*, *E .polecki*, *E. hartmanii*). *E. moshkovskii*, *E. dispar* and *E. histolytica* grouped into distinct clusters. This grouping was evidence of correct species identification and confirmation of the PCR assay results.

There are a number of reasons why it is important to establish the molecular epidemiology of members of the *Entamoeba* complex in Kenya, the most immediate being to accurately treat in endemic areas like Kenya. This report highlights the need for continued epidemiology and PCR-based testing on a larger sample set to establish the burden of *E. moshkovskii* in the Kenyan population and monitor the patterns of infection.

## Data Availability

All data generated or analysed during this study are included in this published article.
